# Characteristics of the Occlusal Plane Associated with Unilateral and Bilateral Articular Eminence Inclination: A Cross-Sectional CBCT Study

**DOI:** 10.3390/dj12100316

**Published:** 2024-09-29

**Authors:** Fátima Erandi Camacho-Álvarez, Silvia Paulina Martínez-Contreras, Jacqueline A. Rodríguez-Chávez, Gerardo Martínez-Suárez, Álvaro Edgar González-Aragón Pineda, Ronald Roossevelt Ramos-Montiel, Carla Monserrat Ramírez-Martínez, Sergio Sánchez-García, Luis Pablo Cruz-Hervert, María Eugenia Jiménez-Corona

**Affiliations:** 1 Especialidad de Ortodoncia y Ortopedia Maxilar, Universidad Cuauhtémoc, San Luis Potosí 78290, Mexico; a20220080@ucslp.net (F.E.C.-Á.); paulina@ucslp.net (S.P.M.-C.); 2 Departamento de Clínicas Odontológicas Integrales, Centro Universitario de Ciencias de la Salud, Universidad de Guadalajara, Guadalajara 44340, Mexico; 3 Departamento de Estomatología, Servicio de Ortodoncia, Hospital Infantil de México “Federico Gómez”, Ciudad de México 06720, Mexico; drsgema@yahoo.com.mx; 4 Facultad de Estudios Superiores (FES) Iztacala, Universidad Nacional Autónoma de México, Tlalnepantla de Baz 54090, Mexico; alvaroedgar@unam.mx; 5 Unidad Académica de Salud y Bienestar y Unidad de Posgrado, Universidad Católica de Cuenca, Cuenca 010107, Ecuador; rramosm@ucacue.edu.ec; 6 División de Estudios de Posgrado e Investigación, Facultad de Odontología, Universidad Nacional Autónoma de México, Ciudad de México 04510, Mexico; c.d.carlarmzmtz@hotmail.es (C.M.R.-M.); sergio.sanchezga@imss.gob.mx (S.S.-G.); 7 Unidad de Investigación Epidemiológica y en Servicios de Salud, Área Envejecimiento, Centro Médico Siglo XXI, Instituto Mexicano del Seguro Social, Ciudad de México 06720, Mexico; 8 Departamento de Epidemiología, Instituto Nacional de Cardiología Ignacio Chávez, Ciudad de México 14080, Mexico

**Keywords:** articular eminence, TMJ, occlusal plane, cone beam computed tomography

## Abstract

The characteristics of the temporomandibular joint (TMJ) are essential in orthodontic and prosthetic treatments. Previous studies have suggested an association between articular eminence inclinations (AEI) and occlusal plane characteristics using radiographs, but no bilateral analysis has been conducted using cone beam computed tomography (CBCT). **Objective:** This study aimed to investigate the specific characteristics of the occlusal plane inclinations associated with unilateral and bilateral AEI using CBCT. **Methods:** We conducted a cross-sectional study to evaluate 200 temporomandibular joints (TMJs) from 100 records obtained at the orthodontic department. We evaluated the association between the AEI, and occlusal plane characteristics like the cant of the occlusal angle, occlusal plane angles, inclination of the upper incisor to the Frankfort plane and palatal plane using both bivariate and multivariate analyses both unilaterally and bilaterally. **Results:** Our findings suggested statistically significant associations (*p* < 0.050) between AEI (bilateral) and occlusal inclination parameters, including the cant of the occlusal plane (Coef. −0.38; 95%CI −0.70:−0.06; *p* = 0.017), occlusal plane angle (Coef. −0.39; 95%CI −0.740:−0.05; *p* = 0.024), and position of the upper incisor relative to the palatal plane (Coef. −0.34; 95%CI −0.63:−0.06; *p* = 0.016). **Conclusion:** This study suggests an association between dental inclinations and AEI, which reflects the anatomical characteristics of TMJ and its related dental structures.

## 1. Introduction

The occlusal plane and the articular eminence inclination play crucial roles in the function and stability of the temporomandibular joint (TMJ) [1,2]. Understanding the relationship between these two factors is important for diagnosing, planning and rehabilitating patients with and without temporomandibular joint disorders (TMDs). Temporomandibular disorders (TMDs) affect the temporomandibular joint (TMJ) and the mandibular muscles, representing a common cause of non-dental orofacial pain, impacting between 5% and 56% of the population. Symptoms include pain, restricted mandibular movement, and joint sounds [3,4,5]. The etiology of TMD is multifactorial, complicating both diagnosis and treatment [3,4,5]. The study of articular eminence inclination and its relationship with the occlusal plane is crucial for understanding TMJ biomechanics and enhancing clinical management, underscoring its importance in orthodontics and prosthetic rehabilitation [3,4,5]. The statics and dynamics of the anatomical characteristics of the TMJ are complex; however, articular eminence is considered one of the key anatomical features of the TMJ [6,7,8,9].

The articular eminence inclination (AEI), located above the temporal bone, represents the anterior part of the glenoid fossa over which the condyle–disc complex slides during various mandibular movements. The AEI is defined as the angle between the articular eminence and the horizontal Frankfort plane. The more pronounced the articular eminence is, the more difficult it is for the condyles to strain downward as they move forward [6,7,8]. This results in greater vertical movement of the condyles, mandibular body, and mandibular arch during opening [6,7,8]. Even though the harmony of the condylar guide is multifactorial, the previous guide and the inclination of the occlusal plane maintain the structural health of the system.

The occlusal plane plays a significant role in determining the functional and aesthetic aspects of dentition. The orientation of the occlusal plane affects the intercuspal position and stability of the masticatory system [10,11,12]. Moreover, it has been found to have a direct correlation with articular eminence inclination [6,7,8].

Several studies have established a relationship between the occlusal plane and the articular eminence inclination, emphasizing the impact of their alignment on the stability and function of the temporomandibular joint [6,8,12,13]. Understanding this relationship is crucial for dental professionals in diagnosing and planning treatment for patients with temporomandibular joint disorders.

There are numerous benefits of using cone beam computed tomography (CBCT) technology in dental and medical imaging. It provides detailed 3D images, reduced radiation exposure, improved treatment planning, precise implant placement, and improved diagnosis of conditions such as impacted teeth or jaw disorders [9,14]. The use of cone beam computed tomography has been validated as a reliable and effective method for evaluating the temporomandibular joint, providing detailed three-dimensional images for accurate assessment [9].

Despite advancements in the understanding of the temporomandibular joint, significant research gaps remain. The methodological variability and complexities in conducting comprehensive clinical studies have left the biomechanical characteristics of dental trauma and their effect on adjacent tissues partially unexplored [6,14,15]. While some studies suggest multifactorial etiologies behind TMJ disorders, the precise anatomical predispositions are yet to be fully delineated [9]. The specificity of condylar morphology and its relation to TMD warrants more detailed investigation. Research to date has not fully revealed how articular eminence inclination influences TMJ functionality or the onset of TMD [6,8,9]. Moreover, no study has performed a multivariate analysis of the associations between these anatomical features on an individual basis [6,8]. Additionally, while it is recognized that joint adaptation and characteristics are not always bilateral or symmetrical, there is an absence of studies evaluating the influence of associated factors on the AEI in a separated bilateral manner and as a whole, leaving a gap in our bilateral understanding of these articular variations [8].

In this study, we aimed to investigate the specific characteristics of the occlusal plane associated with unilateral and overall articular eminence inclinations using cone beam computed tomography. Our hypothesis was that a steeper slope of the articular eminence leads to increased forward and downward movements of the mandibular condyle when opening the mouth. This altered movement pattern may affect the biomechanics of the temporomandibular joint and potentially increase its range of motion. By studying these characteristics, we aimed to improve our understanding of the anatomical features of the temporomandibular joint and their impact on diagnosis and treatment, which could significantly contribute to the development of more precise diagnostic and treatment planning protocols for temporomandibular joint disorders, ultimately enhancing patient care quality.

## 2. Materials and Methods

This was a cross-sectional study. We randomly selected 100 CBCT images of patients from 2021 to 2023 from the radiology department of the postgraduate course in Orthodontics and Maxillary Orthopedics at the Cuauhtémoc University of San Luis Potosí.

### 2.1. Selection Criteria

Our inclusion criteria were as follows: (1) adult patients between 18 and 50 years of age at the time of CBCT registration [16]; although maxillofacial growth typically ceases by age 21 [17], the occlusal plane and joint anatomy can still be altered by factors such as loss of vertical dimension, bruxism, parafunctional habits, sex or changes in bone density [8]; (2) no evidence of prior orthodontic treatment, such as braces and fixed or removable retainers; (3) no evidence of missing teeth; and (4) no evidence of craniofacial syndromes. The exclusion criterion was CBCT with image alterations that impaired the identification of complete anatomical and facial structures, as well as the placement of the bite block in the CBCT measurement.

### 2.2. Data Acquisition

A NEWTOM VGi CT scanner was used with the following parameters: 110 kV, 1–20 mA pulsed mode, 18–26 s scanning time, and a large FOV of 15 × 15 cm. During the CBCT examination, the patient was standing, the head was oriented with the Frankfort plane (Po-Or) parallel to the floor and perpendicular to the sagittal midline previously located in the axial plane, connecting the opisthion (Op) and crista galli (Cg), and a chin support was used. Patients were clearly informed about the radiological procedure and were asked to avoid any kind of movement and maintain centric occlusion. After the X-ray scan, the DICOM image files were analyzed by using 3D Slicer Software version 5.2.2 [18] (open source, http://www.slicer.org. accesed on 1 February 2024).

### 2.3. Tridimensional Reference Planes

**Figure 1:**
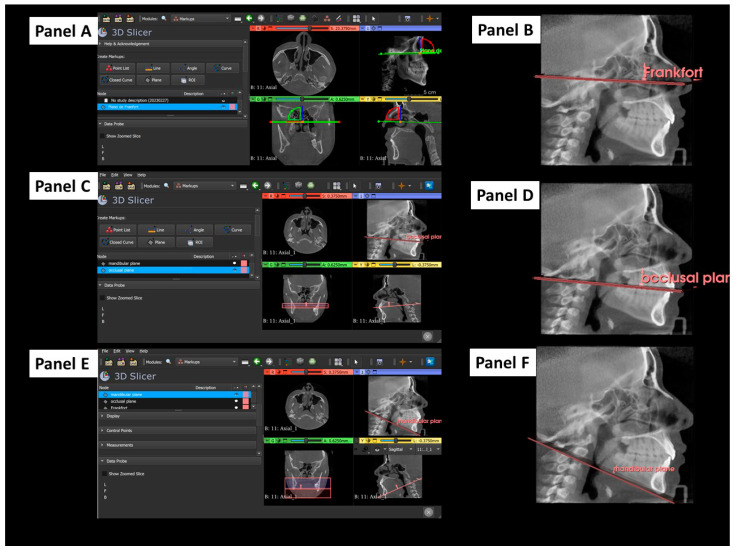
Cephalometric 3D landmarks in CBCT using 3D Slicer software. Panel (**A**): selection of the Frankfort plane. Panel (**B**): visualization of the Frankfort plane from the sagittal view. Panel (**C**): selection of the occlusal plane. Panel (**D**): visualization of the occlusal plane from the sagittal view. Panel (**E**): selection of the mandibular plane. Panel (**F**): visualization of the mandibular plane from the sagittal view.

The 3D Frankfort horizontal reference plane is defined by bilateral landmarks, specifically the porion (the upper rim of the external auditory meatus) on both the left and right sides and the orbitale (the lowest point on the inferior margin of the orbit), which are also on both sides [2,13,19] (Figure 1A,B).

The 3D occlusal plane is defined by tracing a plane through three specific points: the most prominent point of the incisal edge of the upper incisor and the points of the distobuccal cusps of both upper first molars. These points were identified in study models or in 3D reconstructions from CBCT scan data. Using specialized software for 3D cephalometric analysis, a plane was traced to connect these three points to represent the patient’s 3D occlusal plane (Figure 1C,D).

The 3D mandibular plane is traced by connecting three points: the left and right gonions (Go), the most posterior and inferior points of the mandible, and the menton (Me), the most inferior point of the mandibular symphysis (Figure 1D,E).

### 2.4. Outcome and Independent Variables

Articular eminence inclination is defined as the angle formed by the slope of the articular eminence in relation to the 3D Frankfort horizontal plane. This indicates the degree to which the eminence is pronounced or flat. The angle along the posterior slope of the articular eminence was assessed by calculating its inclination within sagittal sections for both the right and left sides [13,19,20].

**Figure 2:**
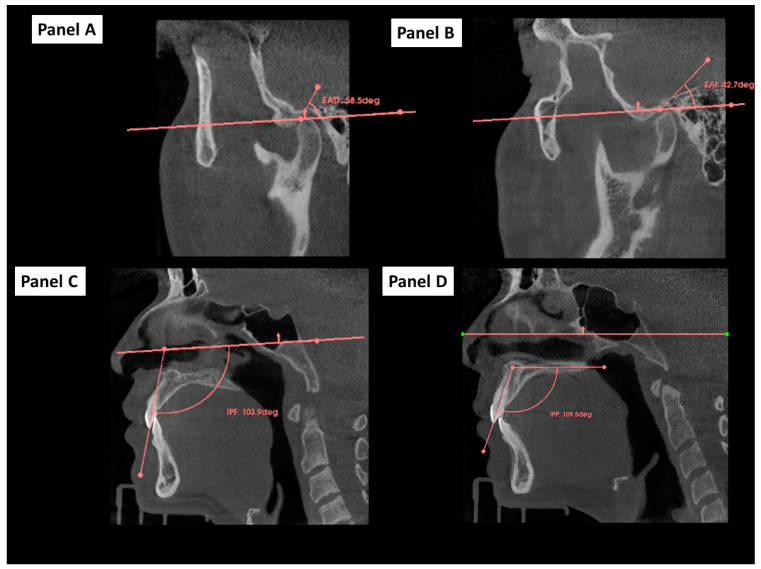
Determination and measurements of 3D angles in CBCT using 3D Slicer Software. Footnote: Panel (**A**): measurement of the left articular eminence angle (EAD). Panel (**B**): measurement of the right articular eminence angle (EAD). Panel (**C**): measurement of the upper incisor angle relative to the Frankfort plane. Panel (**D**): measurement of the upper incisor angle relative to the palatal plane.

Three-dimensional occlusal cant angles were measured as the angle between the 3D occlusal plane and a horizontal reference plane, such as the 3D Frankfort horizontal (FH) plane or the sella–nasion (SN) plane [21,22]. The 3D occlusal plane angle to the mandible was defined as the angle formed between the 3D occlusal plane and the 3D mandibular plane in a three-dimensional cephalometric analysis. Three-dimensional values were obtained for the upper incisor relative to the Frankfort plane and the upper incisor relative to the palatal plane (Figure 2C,D).

### 2.5. Cephalometric 2D Variables

**Figure 3:**
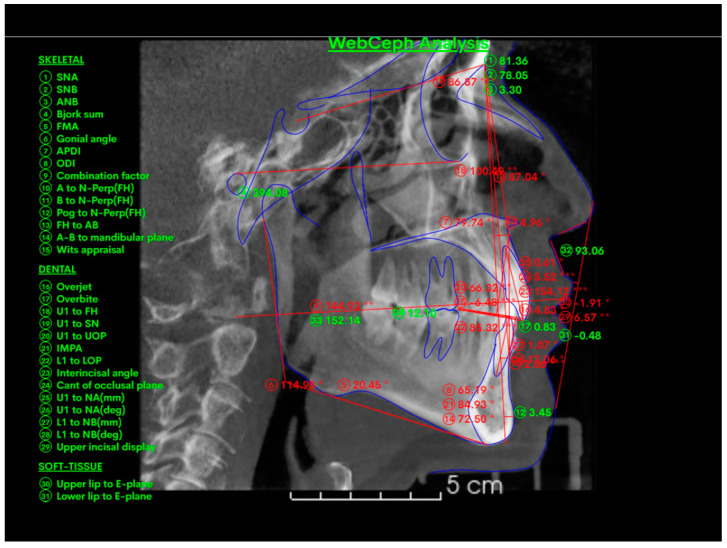
Cephalometric measurements in 2D. Footnote: cephalometric measurements in 2D were performed on a lateral radiograph derived from cone beam computed tomography (CBCT) from an orthogonal view, free of distortion. Using the Webceph program, values were obtained for the interincisal angle, skeletal class, overjet, and overbite. The measurements marked with * indicate ±1 clinical deviation from the norm, while ** indicates ±2 clinical deviations from the norm.

Cephalometric measurements in 2D were performed on a lateral radiograph derived from cone beam computed tomography (CBCT) from an orthogonal view, free of distortion. Using the Webceph program, values were obtained for the interincisal angle, skeletal class, overjet, and overbite (Figure 3).

### 2.6. Population Study

The study population consisted of Mexican adult patients who underwent cone beam computed tomography (CBCT) before the initiation of orthodontic treatment at the Orthodontics Specialty Clinic of Universidad Cuauhtémoc, San Luis Potosí campus, Mexico. CBCT scans were randomly selected from a database covering the period from 2021 to 2023. All patients were Mexican.

### 2.7. Sample Size Calculation

We calculated the sample size using an online tool, an a priori sample size calculator for multiple regression [23]. This calculator determines the minimum required sample size for a multiple regression study based on the desired probability level, number of predictors in the model, anticipated effect size, and desired statistical power level. For our study, we set the anticipated effect size (f^2^) to 0.20, the desired statistical power level to 0.8, the number of predictors to 10, and the probability level to 0.05. The calculation indicated that the minimum sample size required for our analysis was 91 participants. Additionally, we considered a 10% increase to account for potential complications in CBCT evaluation.

### 2.8. Randomization

The radiology department of the university obtained an anonymized list of all CBCT records performed during the study period (2021–2023), arranged chronologically. Using the Epidat 4.0 software, a list of 100 random numbers was generated according to the calculated sample size to select the images.

### 2.9. Statistical Analysis

#### 2.9.1. Descriptive Statistics

We calculated the means and standard deviations for variables with a normal distribution, while for those with a non-normal distribution, we reported the median and interquartile range. The Shapiro–Wilk test was used to evaluate the normality of the variables, with a *p*-value > 0.050 indicating a normal distribution. In all analyses, a *p*-value < 0.050 was considered statistically significant.

#### 2.9.2. Multivariate Analysis

Three multivariate linear regression models were constructed: one for the right side, one for the left side, and a bilateral model that included both measurements, identified as belonging to the same individual (cluster specification), to account for potential bias due to the lack of independence when evaluating the right and left sides together. In these multivariate models, the dependent variable was articular eminence inclination, while the independent variables were the cant of the occlusal plane and occlusal plane inclination. The adjustment variables included the following: Frankfort plane incisor, palatal plane incisor, interincisal angle, overjet, overbite, and age.

The regression models were constructed using the stepwise method, including variables with a Wald statistic value (*p*-value) of 0.200 or less. Although no variables exceeded the established significance level, they were retained in the model due to their theoretical relevance.

All the multivariate linear regression models were tested and met all assumptions of the statistical models. The evaluation of residuals was thoroughly conducted, and key assumptions such as homoscedasticity, multicollinearity, and independence of residuals were assessed using standard diagnostic tests, including variance inflation factors (VIFs) and residual plots.

### 2.10. Ethical Approval and Consent to Participate

The study was approved in 2024 by the UCSLP Ethics and Research Committee (No. CEI-UCSLP-24-004). In compliance with ethical standards, this study utilized anonymized data from an image repository at a radiological center affiliated with the Cuauhtémoc University of San Luis Potosí. The anonymization process involved the removal of all personal identifiers, ensuring that individual data could not be linked to specific persons. The images were processed by an authorized employee under a confidentiality agreement, reaffirming that they lacked identifiable personal information. Given that the images are fully anonymized and the manuscript contains no identifiable details pertaining to individuals, the requirement for consent to publish these images may be waived according to ethical guidelines.

This plane represents the actual occlusal surfaces of the upper and lower teeth more accurately than the 2D occlusal plane traced on a conventional lateral cephalometric radiograph. Evaluating the inclination and orientation of the 3D occlusal plane in relation to other cephalometric reference planes, as well as relevant angular and linear measurements, allows for more accurate diagnosis and treatment planning.

## 3. Results

We selected 100 CBCT images and analyzed 200 joints. In total, 63% were women (n = 63), and 37% were men (n = 37). Their ages ranged from 18 to 50 years, with a mean age of 29.6 years (standard deviation [SD] ± 8.41) (Table 1).

According to the skeletal class, 9 were class I, 84 were class II, and 7 were class III. The variables of the left articular eminence, right articular eminence, cant of occlusal plane, occlusal plane, Frankfort plane upper incisor, palatal plane upper incisor, interincisal angle, overjet, and overbite are described in Table 1.

### 3.1. Right Articular Eminence Inclination

According to the bivariate regression model, only the angle of the occlusal plane to the Go–Me (Coef = −0.39; 95%CI = −0.79, −0.01; *p* = 0.048) was significantly associated with the inclination of the right articular eminence, indicating that as the angle of the occlusal plane to the Go–Me increases, the inclination of the right articular eminence decreases. It is important to highlight that the *p*-value is borderline (close to *p* = 0.050), and the confidence interval is close to zero, meaning the effect on the inclination of the articular eminence may not be substantial in bivariate analysis. However, it is necessary to base interpretations on the multivariate model rather than the bivariate model. No significant association was found for the cant of the occlusal plane. Moreover, among the covariates, only Class II malocclusion showed statistical significance (Coef. = 7.89; 95%CI: 1.40 to 14.39; *p* value = 0.018), suggesting that Class II malocclusion is positively associated with the inclination of the right articular eminence, as shown in Table 2.

According to the multivariate regression model, the occlusal plane of the Go–Me (Coef. = −0.58; 95%CI = −0.98, −0.17; *p* value = 0.006) was significantly associated with the inclination of the right articular eminence, indicating that for each degree increase in the occlusal plane to the Go–Me angle, there was a decrease of 0.27° in the inclination of the right articular eminence. No significant association was found for the cant of the occlusal plane. Similar to the bivariate model, the only adjustment variable that showed a significant association was Class II malocclusion (Coef. = 10.02; 95%CI = 3.02 to 17.02; *p* value = 0.005), suggesting that for Class II malocclusion, the inclination of the right articular eminence increases by 10.02 degrees, as shown in Table 2.

The comparison between the bivariate and multivariate regression analyses shows how controlling for multiple variables simultaneously affects the values. According to the bivariate model, there was a stronger association between the occlusal plane and Go–Me and the inclination of the right articular eminence (Coef = −0.39) than according to the multivariate model (Coef = −0.58). This suggests that when considering other variables, the regression coefficient becomes lower (more negative) and increases the significant *p*-value (Table 2).

The regression coefficient for Class II malocclusion increased from 7.89 in the bivariate analysis to 10.02 in the multivariate model, indicating a stronger positive association with the inclination of the right articular eminence when other factors are considered. This highlights the importance of multivariate analysis in understanding the relationships between variables by addressing potential confounders (Table 2). However, in both the bivariate and multivariate analyses, the 95%CI is wide, though statistically significant, in contrast to Class I. This could be attributed to unassessed clinical characteristics. Although the inclination is greater in Class II, it is difficult to draw definitive conclusions due to the wide 95%CI, and thus, the results should be interpreted with caution.

### 3.2. Left Articular Eminence Inclination

According to the bivariate analysis of the inclination of the left articular eminence, no effect of the occlusal plane or adjustment variables on the inclination of the articular eminence was observed (Table 3).

However, in multivariate analysis of the inclination of the left articular eminence, significant effects were observed. Specifically, the occlusal plane cant had a significant effect (coefficient (Coef.) = −0.47; 95%CI = −0.79 to −0.16, *p* value = 0.004). Additionally, there were significant effects from the upper incisor relative to the palatal plane (Coef. = −0.37; 95%CI = −0.69 to −0.06, *p* value = 0.020), overbite (Coef. = −0.85; 95%CI = −1.51 to −0.18, *p* value = 0.013), Class II (Coef. = 6.57; 95%CI = 2.85 to 10.29), and Class III (Coef. = 10.62; 95%CI = 1.60 to 19.65, *p* value = 0.013). These results highlight the importance of these variables in influencing left articular eminence inclination (Table 3).

### 3.3. Bilateral Articular Eminence Inclination

According to the multivariate linear regression model analyzing both sides of the eminence, a negative relationship was noted between the cant of the occlusal plane (Coef. = −0.39; 95%CI = −0.70, −0.07; *p* = 0.017), and the occlusal plane to the Go–Me angle (Coef. = −0.40; 95%CI = −0.74, −0.05; *p* = 0.024) with inclination of the articular eminence. In practical terms, this suggests that for every one-degree increase in both the cant of the occlusal plane and occlusal plane to the Go–Me angle, there is a decrease in inclination of approximately 039°. Table 4 shows the correlation of the right and left articular eminence (Table 4).

Additionally, the angle of the upper incisor relative to the palatal plane and overbite showed a negative association. This means that for each degree of increase in the angle of the upper incisor relative to the palatal plane, the inclination of the articular eminence decreases, and for each millimeter increase in the overbite, the inclination decreases.

Compared with Class II malocclusions (Coef. = 8.45; 95%CI = 3.65 to 13.25; *p* value = 0.001) and Class III malocclusions (Coef. = 10.60; 95%CI = 1.03 to 20.03; *p* = 0.001), Class II malocclusions had a positive effect on the inclination of articular eminence, indicating increases of approximately 8 degrees and 10 degrees for Class II and III malocclusions, respectively. No significant differences were observed between the right and left sides of articular eminence inclination (Table 4).

## 4. Discussion

In our study, we found that both the cant of the occlusal plane and the angle from the occlusal plane to the Go–Me negatively impacted the inclination of the articular eminence, whereas Class II and Class III malocclusions positively influenced it. Importantly, there was no consistency in the factors affecting both sides, but bilateral, occlusal plane factors and other adjustments had a significant effect. Based on these results, we accept our hypothesis that variations in the occlusal plane are significantly associated with changes in the inclination of the articular eminence, influencing the movement of the mandibular condyle.

Our study focused on determining whether there is a relationship between articular eminence and inclination of the occlusal plane to highlight the importance of managing this relationship in joint problems. This is crucial because determining this inclination is necessary to offer a more comprehensive diagnosis and treatment plan in orthodontic cases, orthognathic surgery, and complete prosthetic rehabilitation, among other treatments. The correlations we found between both joints and the inclination of the occlusal plane and the inclination of the upper incisor to the palatal plane and between the inclination of both joints and overbite height emphasize the importance of the position of the teeth in joint function [24,25,26]. Specifically, it relates the inclination of the upper incisor to its bone base and the distance it covers during mandibular movements in relation to the temporomandibular joint [11,19,21,24,25,27].

Notably, our findings revealed a relationship between the right articular eminence, inclination of the occlusal plane, and age [2,13]. Additionally, a 1° increase in the inclination of the upper incisor relative to the Frankfort plane (FP) resulted in a 0.16° increase in the inclination of the left articular eminence [2,6,12]. Conversely, a 1° increase in the inclination of the upper incisor relative to the palatal plane resulted in a 0.26° decrease in the inclination of the left articular eminence [2,12,13]. Similar correlations were observed for the right articular eminence [6].

Over time, many authors have determined the inclination of the articular eminence in different populations by using various imaging techniques. Studies using panoramic and/or transcranial radiographs, such as those by Chiang et al. [28], Rent et al. [29], and Ichikawa et al. [24,30], reported inclinations of 37.7°, 64.4°, and 58.4°, respectively. Other studies using magnetic resonance imaging (MRI), such as that by Ozkan et al. [6], reported inclinations of 43.12°, 60 68.7° and 54.9°. However, MRI does not provide detailed visualization of bone structures that is achievable with cone beam volumetric tomography (CBCT) [31].

CBCT provides accurate and reliable linear measurements for the reconstruction and imaging of dental and maxillofacial structures and has demonstrated superior results for TMJ imaging [9,20]. Dentistry is considered one of the most accurate methods for evaluating bone structures. Therefore, in the present study, CBCT was used to ensure greater certainty in the bone structures, considering the inclusion and exclusion criteria, and to obtain a sample of 100 patients (200 temporomandibular joints) from the tomographic records of the Radiology Service of the Orthodontic Postgraduate Program of Cuauhtémoc University.

We also considered other important variables, such as the inclination of the upper incisor relative to the FP and palatal planes. Individually, these variables did not show statistically significant differences, but in the multivariate analysis, they demonstrated a consistent correlation with the inclination of the articular eminence and occlusal plane. These results suggest that anatomical structures, such as the articular eminence, should be related to the palatal plane without overlooking dental inclination.

Previous publications reported a similar correlation, such as that of Han et al. [32], who found a significant correlation between the incisal guide angle, angle of the occlusal plane, and functional variation in the shape of the temporomandibular joint. Studies on anterior guidance highlight the importance of the relationship between the articular eminence and the position of the upper incisor with the FP to achieve adequate anterior guidance and establish a balance in the stomatognathic system.

Our findings regarding the inclination of the temporomandibular joint with the inclination of the upper incisor relative to the FP and palatal plane suggest implementing CBCT studies to analyze the relationship between the bone and dental structures for successful treatment planning. By adopting this measure, practitioners can improve the predictability of tooth movement, ensure treatment stability, and mitigate the risks associated with joint problems. However, it is important to consider the risk of radiation exposure, which is greater than that of panoramic X-rays. CBCT should be used judiciously, considering alternative imaging options that provide adequate information while minimizing radiation exposure and always prioritizing patient safety.

### 4.1. Limitations

Despite the insights provided, our study has limitations, including its reliance on cross-sectional data, which precludes the ability to infer causality. Additionally, the scarcity of literature related to these specific relationships in cone beam volumetric tomography (CBCT) indicates the need for more extensive databases to validate these findings. There is limited information on the relationship between these factors in CBCT, even though they have been studied individually.

Another limitation lies in the fact that the articular eminence is subject to functional loads derived from chewing forces, which can affect its morphological shape. This variability may have influenced our results, as changes in the shape of the articular eminence could impact the accuracy of the measurements taken in this study.

Although CBCT measurements provide valuable information regarding the relationship between the articular eminence and occlusal plane inclination, it is necessary to recognize that other factors, such as posture and airways, also play a crucial role in stimulating and guiding growth. These aspects were not analyzed in this study. Furthermore, future research should evaluate additional variables, including habits, the absence of teeth, and the morphology of the mandibular fossa, all of which can influence the inclination of the articular eminence.

However, to the best of our knowledge, this is the first study to estimate the sample size before conducting the study and to use simple random selection. Additionally, we utilized a multivariate approach to evaluate the associations between articular eminence inclination and occlusal plane factors, both per side and bilaterally.

The external validity of this study is influenced by the sample size and specific characteristics of the participants. The population in this study consists of a particular group of Mexican patients seeking orthodontic treatment at a university clinic, which may limit the applicability of the results to broader populations. Caution is advised when interpreting these findings, and further research with larger and more diverse samples is necessary to confirm the results in different clinical contexts.

### 4.2. Implications

Clinicians may leverage these insights to optimize the diagnostic process and treatment outcomes of orthodontic and prosthetic care. This study underscores the importance of considering the anatomic and functional aspects of the inclination of the articular eminence when assessing temporomandibular disorders.

## 5. Conclusions

Our study suggested that both the cant of the occlusal plane and the angle from the occlusal plane to the Go–Me negatively impact the inclination of the articular eminence, while Class II and Class III malocclusions positively influence it. There was no consistency in the factors affecting both sides, but bilateral, occlusal plane factors and other adjustments had significant effects. These findings support our hypothesis that variations in the occlusal plane are significantly associated with changes in the inclination of the articular eminence, thereby influencing the movement of the mandibular condyle. This highlights the importance of managing occlusal plane inclination in joint problems for a comprehensive diagnosis and treatment plan for orthodontics, orthognathic surgery, and prosthetic rehabilitation. Finally, it is important to take the limitations of the study into account and interpret these conclusions within the context of those limitations.

Future research should further explore the relationship between occlusal plane inclination and other factors, such as posture and airways, and evaluate the long-term effects of occlusal adjustments on TMJ initial diagnosis.

## Figures and Tables

**Table 1:** Characteristics of the variables.

	Mean	S.D.
Right articular eminence	38.3	9.5
Left articular eminence	39.4	8.3
Cant of occlusal plane	6.5	5.3
Occlusal plane	19	4.7
Frankfort plane incisor	113.8	14.2
Palatal plane incisor	112.6	14.4
Interincisal angle	124.7	11.1
Overjet	3.4	2.5
Overbite	2.5	2.1
Age	29.6	8.4

S.D. = Standard deviation.

**Table 2:** Bivariate and multivariate right articular eminence.

Right Articular Eminence	Univariate Linear Regression Model		Multivariate Linear Regression Model	
	Coef. (95%CI)	*p* Value	Coef. (95%CI)	*p* Value
Cant of occlusal plane	−0.045 (−0.31; 0.40)	0.802	−0.27 (−0.68; 0.13)	0.189
Occlusal plane	−0.39 (−0.79; −0.01)	0.048	−0.58 (−0.98; −0.17)	0.006
Upper incisor to Frankfort plane (IPF)	−0.028 (−0.16; 0.10)	0.675	0.17 (−0.15; 0.50)	0.300
Upper incisor to palatal plane (IPP)	0.055 (−0.18; −0.07)	0.401	−0.30 (−0.64; 0.03)	0.077
Interincisal angle	−0.032 (−0.13; 0.20)	0.709	−0.04 (−0.21; 0.12)	0.599
Overjet	−0.33 (−0.41; 1.08)	0.382	0.27 (−0.37; 0.92)	0.403
Overbite	−0.048 (−0.94; 0.84)	0.915	−0.70 (−1.49; 0.09)	0.084
Skeletal class				
Class I	Reference	---	Reference	---
Class II	7.89 (1.40; 14.39)	0.018	10.02 (3.02; 17.02)	0.005
Class III	−7.00 (−2.33; 16.34)	0.140	8.80 (−2.98; 20.59)	0.141
Age	−0.198 (−0.42; 0.02)	0.081	−0.20 (−0.43; 0.01)	0.066
Gender	2.639 (−1.25; 6.53)	0.182	2.20 (−1.84; 6.24)	0.283

Footnote: Coef. = linear regression coefficient; 95%CI = 95% confidence interval.

**Table 3:** Bivariate and multivariate analysis of the left articular eminence.

Left Articular Eminence	Bivariate Analysis		Multivariate Analysis	
	Coef. (95%CI)	*p* Value	Coef. (95%CI)	*p* Value
Cant of occlusal plane	−0.25 (−0.56; 0.05)	0.103	−0.47 (−0.79; −0.16)	0.004
Occlusal planet	−0.02 (−0.38; −0.32)	0.866	−0.20 (−0.59; 0.17)	0.289
Upper incisor to Frankfort plane (IPF)	0.01 (−0.10; 0.13)	0.778	0.32 (−0.01; 0.65)	0.050
Upper incisor to palatal plane (IPP)	−0.01 (−0.12; 0.10)	0.800	−0.37 (−0.69; −0.06)	0.020
Interincisal angle	0.16 (−0.13; 0.16)	0.822	0.05 (−0.10; 0.20)	0.497
Overjet	0.27 (−0.37; 0.93)	0.401	0.36 (−0.16; 0.88)	0.179
Overbite	−0.40 (−1.17; 0.37)	0.308	−0.84 (−1.51; −0.18)	0.013
Skeletal class				
Class I	Reference	---	Reference	---
Class II	3.94 (−1.81; 9.70)	0.178	6.57 (2.84; 10.29)	0.001
Class III	6.84 (−1.42; 15.12)	0.104	10.62 (1.60; −19.65)	0.022
Age (years)	0.01 (−0.19; 0.20)	0.941	−0.01 (−0.21; 0.19)	0.926
Gender				
Male	Reference	---	Reference	---
Female	1.32 (−2.09; 4.75)	0.443	0.08 (−3.59; 3.77)	0.962

Coef. = linear regression coefficient; 95%CI = 95% confidence interval.

**Table 4:** Correlation of the right and left articular eminence.

Articular Eminence	Coef.	95%CI	*p* Value
Left side	Reference	---	---
Righ side	1.10	−0.46; 2.68	0.165
Cant of occlusal plane (°)	−0.38	−0.7; −0.06	0.017
Plano occlusal angle (°)	−0.39	−0.74; −0.05	0.024
Upper incisor to Frankfort plane (IPF) (°)	0.24	−0.03; 0.53	0.088
Upper incisor to palatal plane (IPP) (°)	−0.34	−0.63; −0.06	0.016
Interincisal angle (°)	0.01	−0.11; 0.14	0.793
Overjet (mm)	0.32	−0.20; 0.85	0.227
Overbite (mm)	−0.77	−1.39; −0.15	0.015
Skeletal class			
Class 1	Reference	---	---
Class 2	8.45	3.65; 13.25	0.001
Class 3	10.55	1.03; 20.08	0.030
Age (years)	−0.10	−0.29; 0.08	0.265

Footnote: Coef. = linear regression coefficient; 95%CI = 95% confidence interval.

## Data Availability

Dataset is available upon request from the authors.
